# Soluble ST2 Testing: A Promising Biomarker in the Management of Heart Failure

**DOI:** 10.5935/abc.20150151

**Published:** 2016-02

**Authors:** Humberto Villacorta, Alan S. Maisel

**Affiliations:** 1 Universidade Federal Fluminense – Pós-Graduação em Ciências Cardiovasculares, Niterói, RJ – Brazil; 2 University of California – Division of Cardiovascular Medicine, San Diego – USA

**Keywords:** Heart Failure / therapy, Biomarkers, Pharmacological, Receptors, Interleukin, Prognosis

## Abstract

ST2 is a member of the interleukin-1 receptor family biomarker and circulating soluble ST2 concentrations are believed to reflect cardiovascular stress and fibrosis. Recent studies have demonstrated soluble ST2 to be a strong predictor of cardiovascular outcomes in both chronic and acute heart failure. It is a new biomarker that meets all required criteria for a useful biomarker. Of note, it adds information to natriuretic peptides (NPs) and some studies have shown it is even superior in terms of risk stratification. Since the introduction of NPs, this has been the most promising biomarker in the field of heart failure and might be particularly useful as therapy guide.

## Introduction

Heart failure (HF) is a health problem worldwide.^1-3^ In the city of São Paulo, Brazil, HF was responsible for 6.3% of total deaths in the year 2006.^3^ In the DIGITALIS study carried out in the city of Niteroi, Rio de Janeiro State, the prevalence of overt HF in the community in individuals older than 45 years was 9.3%.^4^ Although HF prognosis has improved with the current medical treatment, the sickest patients are often hospitalized and survival is poor.^1-3^ Thus, new strategies to manage such patients are warranted.

Biomarkers have been proved to be helpful in Heart failure. B-type natriuretic peptide (BNP) and N-terminal-proBNP (NT-proBNP) are considered to be the gold-standard tests for the diagnosis of acute HF. However, the prognostic utility of natriuretic peptides is limited and its role in guiding treatment has not yet been clearly established.

A large number of biomarkers have been studied to attempt to fill this gap. ST2, a marker of myocardial fibrosis and remodeling, is a promising candidate that has been successfully added to conventional tools in the management of patients with HF. This report will explore the biology of this system and review the clinical studies with ST2 tests in the field of HF.

## Biology of Soluble ST2

ST2 is a member of the interleukin 1 receptor family, also known as interleukin 1 receptor-like 1 (IL1RL-1).^5,6^ ST2 stands for "suppression of tumorigenicity 2". It was discovered in 1989,^6^ but only in 2002 Weinberg et al.^7^ reported that it could be expressed by cardiac cells in response to myocardial stress, drawing the attention of researchers to a role in the cardiovascular system. ST2 has two main isoforms: transmembrane or cellular (ST2L) and soluble or circulating (sST2) forms.^5^

**Figure 1:**
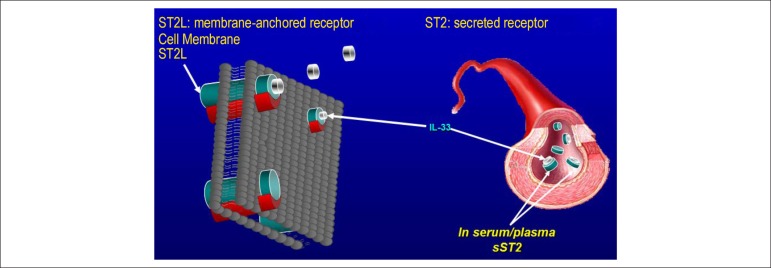
IL-33 interactions with transmembrane receptor, ST2L, and soluble decoy receptor, sST2. The ST2 system acts not only as a mediator of IL-33 function in its ST2L transmembrane isoform (cardioprotective effect) but also as an inhibitor of IL-33 through its soluble sST2 isoform (eliminates the cardioprotective effect).

ST2 is the receptor for interleukin-33 (IL-33), which is an IL-1-like cytokine secreted by living cells in response to cell damage. IL-33 exerts its effects by binding to the transmembrane receptor ST2L isoform. The interaction of IL-33 and ST2L has been proved to be cardioprotective in experimental models, reducing myocardial fibrosis, cardiomyocyte hypertrophy, apoptosis, and improving myocardial function. This cardioprotective action occurs exclusively through the ST2L receptor and not through the soluble receptor. The IL-33/ST2 system is upregulated in cardiomyocytes and fibroblasts in response to cardiac injury. sST2 avidly binds to IL-33 competing with ST2L. The interaction of this soluble receptor with IL-33 blocks the IL-33/ST2L system and, as a result, eliminates the cardioprotective effects described above. Therefore, sST2 is considered a decoy receptor.^8^ Thus, the ST2 system acts not only as a mediator of IL-33 function in its ST2L transmembrane isoform but also as an inhibitor of IL-33 through its soluble sST2 isoform (Figure 1).

Although the main sources of sST2 are cardiac fibroblasts and cardiomyocytes in response to stress or injury, non-myocardial sources are known. Endothelial cells from both macrovascular (aortic and coronary) and cardiac microvascular system are sources of sST2. The contribution of this extracardiac production to the total circulating ST2 and to the pathophysiology of HF is not well established.

ST2 is also associated with inflammatory and immune processes, especially regarding the regulation of mast cells and type 2 CD4 pT-helper cells, and the production of Th2‑associated cytokines. Thus, a role for IL-33/ST2 system has been demonstrated in diseases associated with a predominant Th2 response such as asthma, pulmonary fibrosis, rheumatoid arthritis, collagen vascular diseases, sepsis, trauma, malignancy, fibroproliferative diseases, helminthic infections and ulcerative colitis.^5^,8 As a matter of fact, much of the knowledge on this marker comes from studies on these immune diseases, before the recognition of a cardiovascular role.

## Prognostic Evaluation with sST2 in Acutely Decompensated Heart Failure

Natriuretic peptides (NPs) are the gold standard biomarkers for the diagnosis of HF in patients with acute dyspnea. Although NPs also have a role for prognosis, there is still room for improvement. Other biomarkers may add complementary biological information to NP and increase the prognostic utility in this scenario. Among a great number of new candidates, sST2 is the most promising biomarker according to recent studies. Although not a diagnostic marker, ST2 may be useful in the risk stratification of patients with HF.

In patients with acutely decompensated heart failure (ADHF), the first study to measure ST2 was the Pro‑Brain Natriuretic Peptide Investigation of Dyspnea in the Emergency Department (PRIDE) Study.^9^ In this study, ST2 was measured with an early research-only-use assay (the current Presage ST2 assay is a precise, higher sensitivity method).^10^ In the PRIDE study, 593 patients who presented to the emergency department (ED) with acute dyspnea were included. Levels of sST2 were significantly higher in patients with ADHF than non-HF patients (0.50 vs 0.15 ng/mL, p < 0.001). However, NT-proBNP remained as the best biomarker for the diagnosis of HF.

**Figure 2:**
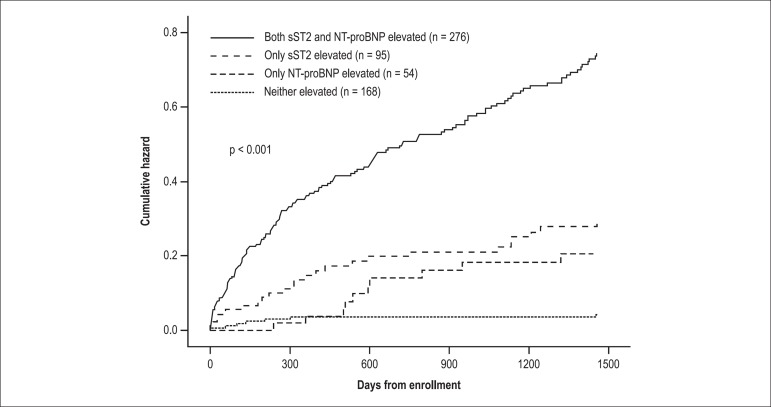
Additive effect of sST2 and NT-proBNP in patients with acute decompensated heart failure. Reprinted with permission.^9,15^

On the other hand, sST2 was a powerful predictor of mortality. Patients who died at 1 year had higher values than survivors (1.08 vs 0.18 ng/mL) and there was a clear association between sST2 levels and mortality rates, with greater concentrations predicting the highest risk. In the multivariate analysis, sST2 remained a strong predictor of 1-year mortality in both patients with and without HF. Of note, the prognostic utility of sST2 added to that of NT-proBNP, such that patients with elevation of both markers had the highest 1-year mortality rate (almost 40%), as depicted in figure 2. This association of sST2 with death emerged soon after enrollment in the study and remained significant out to 4 years from presentation.

Another sub-analysis of the PRIDE Study included 346 patients with the diagnosis of HF.^11,12^ In this study, sST2 concentrations at admission correlated with New York Heart Association functional class, BNP (r = 0.29), NT‑pro‑BNP (r = 0.41), C-reactive protein (r = 0.43), creatinine clearance (r = 0.22), and left ventricular (LV) ejection fraction (r = 0.13). Unlike NPs, sST2 levels did not correlate with age, previous diagnosis of HF, body mass index, atrial fibrillation, or cause of HF (ischemic vs non-ischemic). As observed in the previous study, sST2 was a strong predictor of mortality. In the multivariate Cox regression analysis, sST2 was associated with a 2-fold increase in the risk of mortality regardless of other parameters, including NP. sST2 assessment performed well in HF patients with both reduced (HFrEF) and preserved ejection fraction (HFpEF). Notably, when sST2 values were added in the prognostic model, NT-proBNP was no longer a significant predictor in patients with HFpEF.^13^ It is very important to note the reclassification effect of sST2 over that of NP. High sST2 levels reclassified risk of death in patients with low NP levels. Conversely, in patients with an sST2 value below the median concentration, NT-proBNP > 1,000 pg/mL was not a predictor of 1-year mortality.

In a study by Shah et al.^14^ in 139 patients from the initial PRIDE cohort who had detailed 2-dimensional echocardiography at admission, predictors of sST2 levels in multivariate analysis were right ventricle systolic pressure, LV ejection fraction, LV dimensions (both end systolic and diastolic dimensions), NT-proBNP, heart rate, and jugular venous distension. These data suggest the ST2 biology is involved in the remodeling process, thus affecting the prognosis. As a matter of fact, in this study sST2 level was a predictor of 4-year mortality independent of other traditional clinical, biochemical, and echocardiographic risk markers.

Values of this new and the old assays are not comparable. Thus, using the more sensitive Presage ST2 assay (Critical Diagnostics, San Diego, CA, USA), a value ≥ 35 ng/mL is associated with worse prognosis in patients with HF and this has been the recommended cutoff for this purpose.^15^ However, it is expected that average concentrations of sST2 in ADHF may be greater at the time of presentation. In the PRIDE Study, the median Presage ST2 value in patients with ADHF was 42.7 ng/mL. The values of ST2 in survivors and non survivors at 1 year were 67.4 vs 35.8 ng/mL. Additionally, greater values are expected in patients with more advanced disease. For example, Zilinski et al.^16^ evaluated the role of ST2 in a very sick population with HF. Median concentration of ST2 was 148 ng/mL (interquartile range 88 to 226 ng/mL). Notably, despite these high values, ST2 remained a predictor for death, whereas NT-proBNP, high sensitivity troponin, and renal function were not.

Finally it is noteworthy to comment on the comparison of ST2 measurements with other biomarkers in the setting of ADHF. In a study with 5,306 patients carried out by the Global Research on Acute Conditions Team (GREAT), among a great number of biomarkers measured at admission in patients with ADHF, ST2 emerged as the strongest biomarker with the ability to reclassify death risk beyond a clinical model. ST2 was the best predictor of both 30-day and 1-year mortality.^17^

## Serial Measurement of Soluble ST2 in Patients with Acute Heart Failure

**Figure 3:**
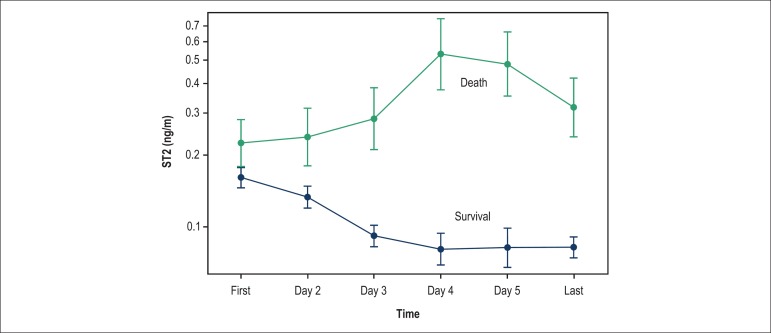
Variation of sST2 values according to survival state in patients hospitalized with heart failure. Reprinted with permission.^19,20^

Although baseline ST2 values at admission have been proved to predict outcomes, serial measurements may be of even greater value. The biological variation and the low index of variation of ST2 make it a good candidate for monitoring and possibly guiding therapy in ADHF.^18,19^ Additionally, sST2 values are not significantly influenced by age, gender, body mass index, and renal function, as opposed to NPs.^19^ One of the first studies to assess serial measurements of sST2 was carried out by Boisot et al.^20^ In this study sST2 was measured on a daily basis in patients admitted with ADHF and demonstrated that this biomarker quickly changes in response to treatment. Patients whose values decreased rapidly after admission had a good short-term outcome, as depicted in Figure 3. In contrast, those with an increase in sST2 values had a high probability of dying at 6 months.

**Figure 4:**
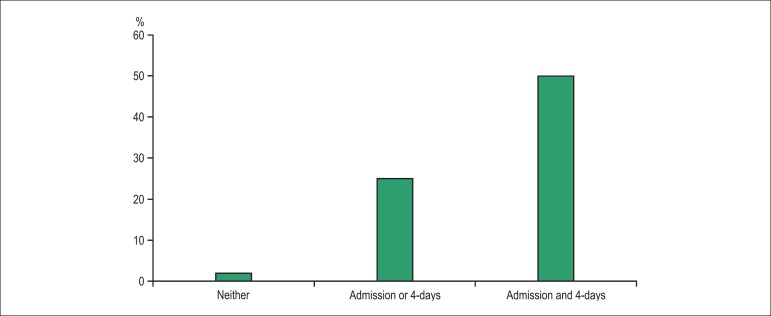
Serial measurement of sST2 in ADHF. Patients with sST2 ≤ 76 ng/mL at presentation and ≤ 46 ng/mL on day 4 had the lowest mortality rate (3%), whereas those with both sST2 values above these cutoff points had the highest mortality (50%)^21^

More recently, similar results were obtained by Manzano-Fernandez et al.,^21^ using the newer Presage assay. They found that median concentrations of sST2 decreased from 62 to 44 ng/mL and those patients with persistent elevation on day 4 had a higher risk of death. Those with both admission and day 4 values above the cutoff had the highest mortality rate in contrast with very low mortality rate when both values were below the cutoff points (Figure 4). Finally, Breidthardt et al.^22^ observed that sST2 values significantly decreased from admission to 48 h, especially in those with favorable outcomes, with a median reduction of 42% in survivors *versus* 25% in non survivors.

It is important to reiterate that in the abovementioned studies, the prognostic value of sST2 was additive or even superior to that of NPs. The dynamic changes in sST2 from admission to discharge and the final value at the end of the hospitalization both contribute to the prediction of long-term prognosis.^9-22^ In chronic HF, ST2 has been shown to predict myocardial remodeling.^23,24^ The association of this biomarker with the remodeling process raises the possibility of identifying those most likely to respond to antiremodeling therapies. For example, in the setting of ADHF, patients with high sST2 levels benefit most from beta-blocker therapy.^21^

## Prognostic Value of Soluble ST2 in Chronic Heart Failure

Consistent with the ADHF data, soluble ST2 has been proven to be useful as a prognostic marker in chronic HF.^25^ The first evaluation in this setting was made by Weinberg et al.,^26^ in a sub-study of Prospective Randomized Amlodipine Survival Evaluation 2 (PRAISE-2). This analysis included 161 patients with class III or IV nonischemic HF and found that serial changes, but not baseline ST2 values, were associated with increased risk for death or transplantation. More recently, Ky et al.^27^ reported data on a larger population of patients with chronic HF. In this multicenter study of 1,141 patients from the Penn Heart Failure Study (PHFS), sST2 and NT‑proBNP were compared with the Seattle Heart Failure Model (SHFM) for the prediction of death or cardiac transplantation at 1 year. The combination of sST2 and NT‑proBNP had a performance similar to that of the SHFM. In terms of assessing individual patient risk, sST2 performed as well as NT-proBNP, but was not superior to SHFM alone. However, adding the two biomarkers to the SHFM score improved risk discrimination by reclassifying 14.9% of patients into more appropriate categories. In contrast with the study by Weinberg et al.,^26^ Ky et al.^27^ found a robust, independent association of a single baseline measure of sST2 and adverse outcomes. According to the investigators, these differences could be due to a larger sample size, a more sensitive sST2 assay, and a broader population with HF.^25^

These initial results were confirmed in the Barcelona Study, where the novel high-sensitivity sST2 assay was used in the assessment of 891 patients at a structured multidisciplinary HF center.^28^ In the multivariate Cox proportional hazard models, sST2 and NT-proBNP significantly predicted death beyond conventional risk factors. Importantly, the net improvement in reclassification after the separate addition of sST2 to the model with established risk factor and NT‑proBNP was a significant 9.90%.

It is noteworthy that in the Barcelona study, the performance of sST2 was not influenced by renal function, as observed with NT-proBNP. The inclusion of sST2 along with other biomarkers improved the prediction in patients with renal failure, even more than in the whole population.^29^

Another additional contribution of the Barcelona study was the comparison of different fibrosis biomarkers. sST2 and galectin-3 are both associated with fibrosis and cardiac remodeling and galectin-3 has been shown to predict outcomes.^30^ Head-to-head comparison of these two biomarkers revealed that sST2 was superior to galectin-3 in risk stratification.^31^ Both markers were associated with increased risk for all-cause mortality, but only sST2 was associated with cardiovascular mortality. Additionally, sST2 significantly refined the discrimination and the reclassification analysis, whereas galectin-3 had minor effects in this regard.

**Figure 5:**
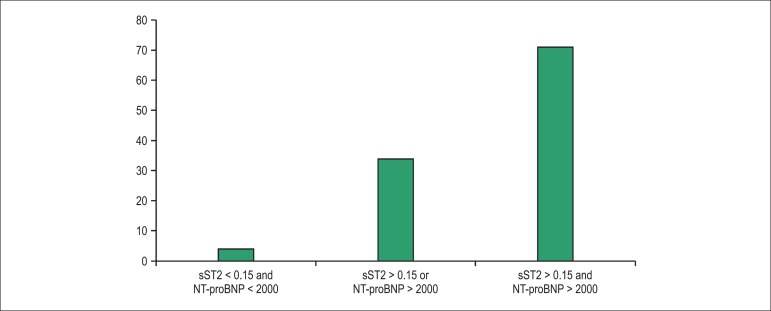
Additive value of sST2 and NT-proBNP in the prediction of sudden death in patients with chronic heart failure.^32^

ST2 has also been shown to be a good predictor of sudden death in patients with mild to moderate systolic HF. In the case-control study Muerte Subita en Insuficiencia Cardiaca (MUSIC), elevation of ST2 and NT-proBNP above the cut-off value was associated with a high rate of sudden death (71%), in contrast with a very low rate (4%) when the two biomarkers were below the threshold (^Figure 5^).^32^ This is an important piece of information considering that, at present, no single test reliably predicts sudden death in patients with HF.

In recent studies, the prognostic value of sST2 in chronic HF has been confirmed. Good performance was observed in the Controlled Trial Investigating Outcomes of Exercise Training (HF-ACTION) study, which was a multicenter randomized study of exercise training in HF,^33^ and in the CORONA study.^34^ Very recently, Gruson et al.^35^ evaluated the value of sST2 in addition to NPs (BNP, NT-proBNP, and proBNP _1-108_) and conventional risk factors such as age, LV ejection fraction, and estimated glomerular filtration rate. sST2 was the strongest predictor of cardiovascular death. In another study, sST2 was also useful and additive to NPs in patients at risk for HF. Daniels et al.^36^ reported on 588 outpatients who were referred for echocardiography. High sST2 levels were independently associated with 1-year mortality, even among the subgroup of 429 patients with no history of HF. Importantly, no patient with an sST2 value below the median levels died in the first 6 months of follow-up.

Taken together, these studies suggest a role for ST2 in the setting of chronic HF, which is additive and in some studies even superior to that of NPs. The 2013 American College of Cardiology and American Heart Association guidelines for the management of HF have, for the first time, made a recommendation for fibrosis biomarkers, such as ST2 and galectine-3, in both acute and chronic HF. They provide a class IIb recommendation and recognize the value of ST2 as a predictor of death and hospitalization. On top of that, the additive prognostic value to that of NPs is emphasized.^37^

## Serial Measurement of ST2 in Chronic Heart Failure

We need to understand the biological variation of a biomarker if it is a candidate to be measured serially. The biological variation of sST2 was recently assessed by Wu et al.,^18^ whose study included 17 healthy subjects over a period of 8 weeks. The variability of the biomarker levels that occurred in the absence of significant clinical instability was assessed. They found that the reference change value for sST2 was 30%, much lower than the one observed with galectin-3 (60%) or NT-proBNP (92%). The index of individuality (a measure to evaluate whether serial measurements add significantly to a single assessment) for sST2 was 0.25, suggesting value from serial measurements. In comparison, the same index for galectin-3 was 1.0, indicating that galectin-3 is useless for serial measurements. These data suggest that sST2 is a potential biomarker for monitoring and possibly guiding therapy in patients with HF.

Three important studies have addressed the value of sST2 serial measurements in chronic HF, all of them using the new Presage assay. The first one is a substudy from the Controlled Rosuvastatin Multinational Trial in Heart Failure (CORONA) Study.^34^ sST2 was measured in 1,449 HF patients and in 1,309 controls; a second sample was available three months after randomization. The median follow-up was 2.6 years and 28.2% reached the primary endpoint of cardiovascular death, nonfatal myocardial infarction or stroke. Median concentration of sST2 at baseline was 17.8 ng/mL (interquartile range 13.0 to 25.0).

After initial adjustments for conventional variables, baseline sST2 was a significant predictor of all endpoints, including the primary endpoint, death, worsening HF, and hospitalization for HF. When NT-proBNP and C-reactive protein were added to the model, sST2 was no longer a predictor of primary outcome, but remained significantly predictive of death from worsening HF, cardiovascular hospitalization, and hospitalization for HF worsening.

In the 1,309 patients with a new measure after 3 months, the overall sST2 variation was minimal (median 0, interquartile range: -3 to 3 ng/mL). However, a few patients did have a change in the biomarker level. Patients who experienced a decrease in sST2 by 3 months had a reduced risk of hospitalization for HF worsening and hospitalization for cardiovascular causes. An increase in sST2 of ≥ 15.5% was associated with hospitalization for cardiovascular causes, but not with any other endpoint on univariate analysis. However, after full adjustments, an increase in sST2 significantly predicted both the primary outcome and hospitalization for cardiovascular causes.

In the pro-BNP Outpatient Tailored Chronic Heart Failure Therapy (PROTECT) study,^38^ of 151 subjects with HF due to LV systolic dysfunction in whom sST2 was measured, 145 patients had more than one sample available for serial assessment. In this study, sST2, highly-sensitive troponin T (HsTnT) and growth differentiation factor 15 (GDF15) were added to a model that included clinical variables and NT-proBNP. At baseline, all three biomarkers improved risk prediction beyond clinical variables, whereas NT-proBNP was no longer a predictor of prognosis.

When measured serially, sST2, but neither HsTnT nor GDF15, changed significantly over a median of 10 months of follow-up as compared with baseline. Using Cox proportional hazard models, baseline sST2 < 35 ng/mL was associated with longer time to first cardiovascular event (HR 0.30, 95% CI 0.14 to 0.63, p = 0.002). Importantly, a change in sST2 values from < 35 to > 35 ng/mL during follow-up was associated with shorter time to cardiovascular event (HR 3.64, 95% CI 1.37 to 9.67, p = 0.009). Of note, sST2 values at 3 and 6 months added significantly to baseline measures for prognostication.

An additional analysis demonstrated that the percentage of time spent below the threshold of 35 ng/mL was one of the strongest predictors of events at 1 year. Additionally, patients were categorized into 3 classes: 1) those whose sST2 values were always < 35 ng/mL; 2) sometimes < 35 ng/mL; and 3) never < 35 ng/mL. A longer period of time with sST2 concentrations < 35 ng/mL predicted a decrease in LV end-diastolic index, suggesting a role for sST2 in LV remodeling surveillance.

Finally, the effects of medications on sST2 serial measurements in the PROTECT study were assessed.^39^ Those with elevated baseline sST2 concentrations who achieved higher beta blocker doses had significantly lower risk of events than those titrated to lower beta blocker doses. Those with low sST2 levels and high beta blocker doses experienced the lowest rate of events.

In the Valsartan Heart Failure Trial (VAL-HeFT), sST2 was measured at baseline, after 4 months and 1 year, in 1,650 patients with LV systolic dysfunction.^40^ In a Cox regression model, baseline sST2 values added significant information regarding first morbid event, death, but no HF hospitalization. The baseline sST2 performance was modest and was displaced by NT-proBNP. However, when analyzed serially, an increase in sST2 concentrations from baseline to 12 months was an excellent predictor of events. When this was added to baseline clinical models, an increase in sST2 values was associated with all outcomes and improved the c-statistics from 0.71 to 0.74. However, decreases in sST2 from baseline to 12 months were not associated with reduced risk of events.

It should also be noted that ACE inhibitors and beta blockers were associated with lower sST2 concentrations, whereas digoxin and diuretics were associated with higher sST2 values. A plausible explanation for the latter finding is the link between sST2 and atrial fibrillation and the association of this biomarker with clinical congestion.^41^

## Future Directions

sST2 may potentially be looked upon as a HgA1C of HF; in other words the sST2 value has inputs from wall stress, inflammation, macrophage activation (fibrosis), as well as a number of still to be determined inputs. Taking these into account, a single sST2 measurement should allow titrating therapy and monitoring the clinical state of the patient. In addition, since sST2 is such a strong marker of the risk of death, it would not be surprising to see a level be used to make decisions when patients are on the cusp of such therapies such as implantable cardioverter defibrillators (ICD), cardiac resynchronization therapy (CRT), CardioMems implantation (pulmonary artery pressure monitoring), and even left ventricular assist device.

## Conclusion

sST2 is a biomarker that has jumped through all the "hoops" expected from a useful biomarker. It is the only new biomarker that can be of value today when caring for patients with both acute and chronic HF. New biomarkers are warranted and have been explored in recent reports.^42,43^ More than one decade ago, NPs emerged as the first biomarkers for the diagnosis of acute HF.^44,45^ Since then, this is the most promising biomarker for the management of such patients, adding to NPs, especially for guiding therapy. Prospective studies testing this hypothesis are more than welcome.
